# Emphysematous Pyelonephritis Associated With Emphysematous Gastritis and Air in the Portal Vein

**DOI:** 10.4021/gr287w

**Published:** 2011-03-20

**Authors:** Jagadish Akella, Gilda-Diaz Fuentes, Sunjeet Kaur, Sindhaghatta Venkatram

**Affiliations:** a Division of Pulmonary Medicine and Critical Care Medicine, Bronx Lebanon Hospital Center, Affiliated with Albert Einstein College of Medicine, Bronx, NY, USA; b Department of Medicine, Bronx Lebanon Hospital Center, Bronx, NY, USA

**Keywords:** Emphysematous pyelonephritis, Emphysematous gastritis, Air in the portal vein, Diabetes mellitus

## Abstract

Emphysematous gastritis with portal venous air is a rare condition usually caused by gas forming organisms. This may be secondary to local spread of an infection through the mucosa or rarely hematogenous dissemination from a distant focus. We present a young diabetic woman with uncontrolled diabetes mellitus who was admitted with sepsis and severe abdominal symptoms. Investigation revealed emphysematous pyelonephritis due to E. coli infection associated with emphysematous gastritis and air in the portal tract. She improved with broad spectrum antibiotics, fluid resuscitation and electrolyte and diabetic management. To our knowledge this is the first report showing the association between emphysematous pyelonephritis and gastritis with air in the portal system.

## Introduction

Emphysematous gastritis with portal venous air is a rare condition. The presence of gas within the parenchyma of solid organs or the walls of hollow viscera may be due to a variety of pathologic or benign entities. Besides infection with gas-forming bacteria, other possible sources include bland tissue infarction with necrosis, enteric fistula formation, and reflux from an adjacent hollow viscous.

Gas associated with infection is generally thought to consist of carbon dioxide and nitrogen secondary to the fermentation of glucose by some species of bacteria. Poor glycolysis at the tissue level in diabetic patients results in increased glucose concentrations within the interstitial fluid. Other clinical factors that contribute to the increased production or slowed removal of gas include a depressed cell-mediated immune response, local tissue necrosis, and the presence of arteriosclerosis [1].

We report a patient with poorly controlled juvenile diabetic presenting with emphysematous pyelonephritis associated with emphysematous gastritis and air in the portal vein.

## Case Report

A 24-year-old Hispanic woman with insulin dependent diabetes mellitus (DM) on insulin pump was admitted with abdominal pain, fever and chills of three days duration. She reported worsening of non-radiating left flank and dull epigastric pain associated with nausea. Her past medical history was remarkable for peripheral neuropathy and gastroparesis due to DM and recurrent episodes of urinary tract infections.

On examination, the patient was alert, oriented and in mild distress. Her initial vitals revealed BP 128/81 mmHg, tachycardia (103 bpm), respiratory rate 16/min, fever 38.2 °C, and saturation of 98% on ambient air. Abdomen was soft with tenderness in the suprapubic, epigastric and left lower quadrant and no peritoneal signs were elicited. Cardiac and respiratory exams were unremarkable. Lower extremity examination revealed decreased sensation and an insulin pump in the left thigh. Urogenital exam was normal with no fistulae or ulcers.

**Figure 1:**
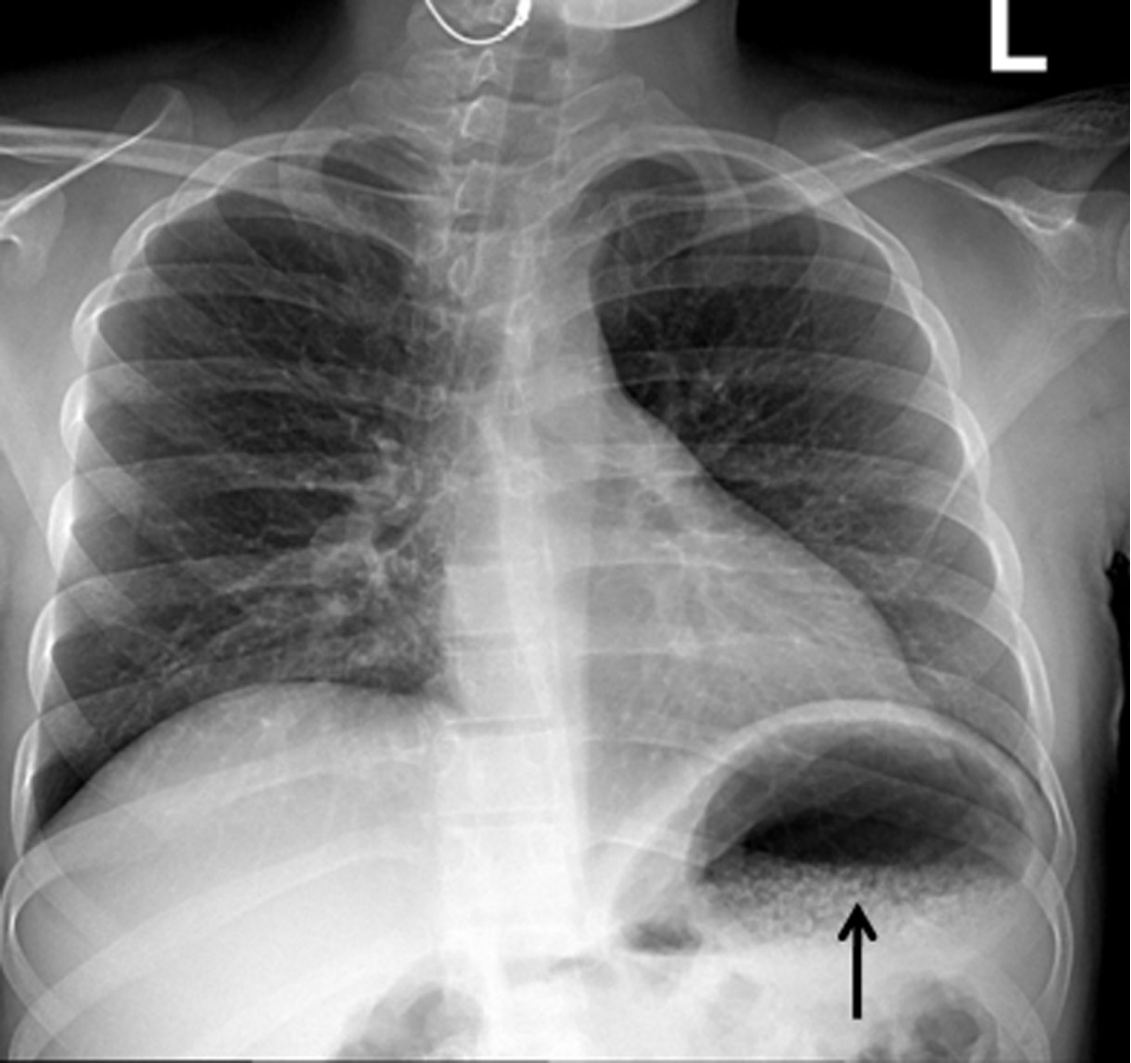
Chest roentgenogram showing innumerable bubbles outlining the stomach in a mottled distribution (arrow).

**Figure 2:**
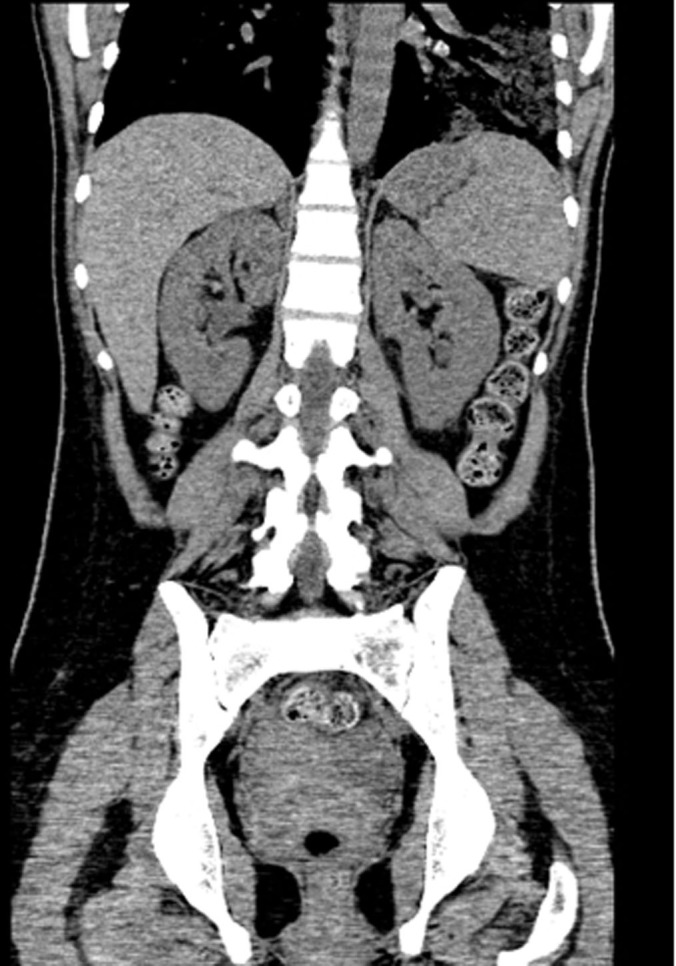
Coronal view of abdominal computed tomogram without contrast showing air in the collecting system of both kidneys.

**Figure 3:**
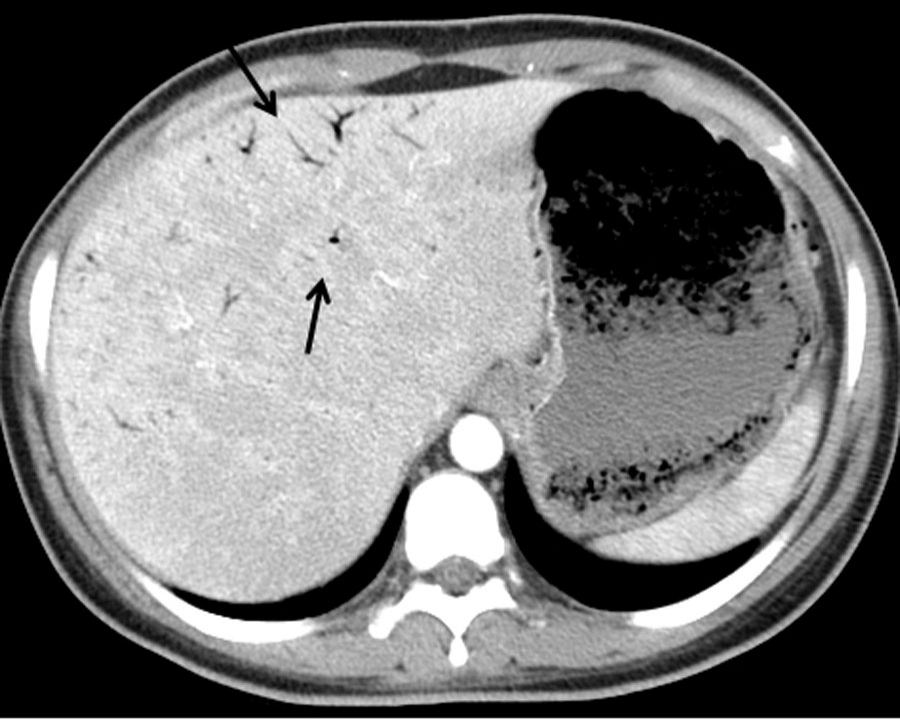
Abdominal computed tomogram with arrows pointing to air in the portal tract and bubbles outlining the stomach.

**Figure 4:**
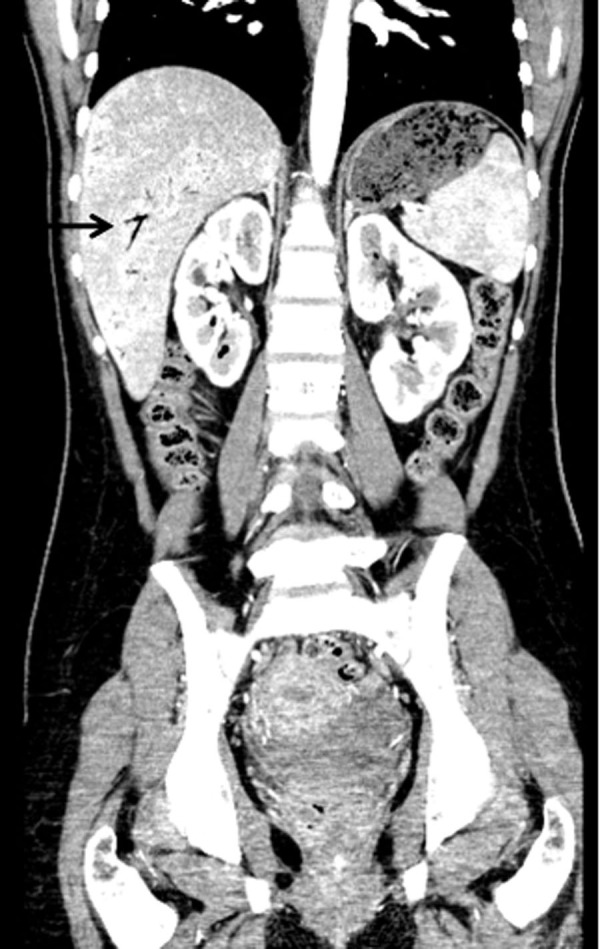
Coronal view of abdominal CT with contrast showing air in the collecting system of both kidneys and air in the portal tract (arrow).

Laboratory showed white cell count of 12.1/mm^3^ with left shift, serum BUN 20 mg/dL and creatinine of 1.3 mg/dL. Urinalysis was positive for large leukocytes and leukocyte esterase. Chest roentgenogram revealed air in gastric wall with food in stomach lumen and no evidence of free air under diaphragm (Fig. 1). Computed tomogram (CT) of the abdomen without contrast showed air in the collecting system of both kidneys (Fig. 2). Subsequent abdominal CT with contrast 12 hours later showed rapid development of gas within the gastric wall, gastric venous drainage and portal venous tree, gas bubbles within the collecting system of both kidneys and urinary bladder with evidence of bilateral pyelonephritis (Fig. 3, 4). The patient was admitted to the medical intensive care unit for emphysematous pyelonephritis with concomitant emphysematous gastritis and air in the portal venous system.

She was treated with intravenous antibiotics (Vancomycin, Fluconazole and Imipenem cilastatin) and hydration. Her blood glucose level was controlled with insulin drip. Emergent esophagogastroduodenoscopy revealed non-bleeding erosive gastropathy with no signs of necrosis. Serial abdominal exams did not show any findings suggestive of acute abdomen. Urine culture grew Escherichia coli sensitive to Imipenem.

The patient improved clinically with medical management. Repeated abdominal CT showed resolution of previously noted gas bubbles within gastric wall, gastroepiploic veins, portal venous tree and collecting system of both kidneys. She was discharged home in stable condition.

## Discussion

Emphysematous gastritis is a rare variant of phlegmonious gastritis caused by gas forming organisms. This may be secondary to local spread through the mucosa or rarely hematogenous dissemination from a distant focus. Emphysematous gastritis is an uncommon disease due to the abundant blood supply, acidic pH and efficient mucosal barrier found in the stomach [2]. Of the reported cases in the literature, caustic ingestion (37%) and alcohol abuse (22%) were found to be the most common causes for emphysematous gastritis; other reported causes include mechanical (instrumentation), pulmonary, ischemic and bacterial sources [2, 3]. There are no predilections with regard to age, sex, or diabetic status.

Clinical presentation can be dramatic with severe abdominal pain, nausea, vomiting, hematemesis, fever and tachycardia [4]. CT abdomen is the radiologic modality of choice for the detection of intramural gas and evaluation for the presence of pneumoperitoneum or portal venous gas. CT may also demonstrate irregular mucosal fold thickening and may be used to monitor response to treatment or disease progression [1, 5].

Early endoscopic evaluation will show a “cobblestone” appearance of the gastric mucosa, representing submucosal blebs of air. Common offending bacteria include *E coli, C welchii,* and mixed infections with *Staphylococcus aureus* [6].

Mainstay of treatment of emphysematous gastritis includes antibiotics, intravenous hydration and supportive care. Surgery is generally not advised unless there are complications like perforation. Prognosis of emphysematous gastritis is variable with a reported mortality rate of 60% to 80% despite early aggressive treatment [7].

It is important to differentiate emphysematous gastritis from gastric emphysema. Patients with gastric emphysema or gastric pneumatosis generally do not present with acute abdomen, and the prognosis is excellent. Gastric fold inflammation and thickening are not present, and the patient is usually asymptomatic with spontaneous resolution expected.

Air in the portal vein or its radicals occurs when intraluminal or bacterial gas enters the porto-mesenteric circulation [8, 9]. Mucosal damage, bowel distention and sepsis are prominent conditions associated with portal venous air. The approach to the patient with portal venous air should be directed to the underlying disease [8].

Emphysematous pyelonephritis (EPN) represents a life-threatening infection of the renal parenchyma with gas-forming bacteria. Underlying poorly controlled DM is present in up to 90% of patients who develop this condition. Clinical presentation includes varying degrees of renal failure, lethargy, acid-base irregularities, and hyperglycemia. Emphysematous pyelonephritis carries an overall mortality rate of approximately 50%; *E coli* is found in approximately 70% of cases. Abdominal radiography may demonstrate gas bubbles overlying the renal fossa or may show a diffusely mottled kidney with radially oriented gas corresponding to the renal pyramids. CT will confirm the presence and extent of parenchymal gas and will often allow identification of the source of obstruction when presents [10, 11].

First-line treatment for EPN is similar to emphysematous gastritis and includes fluid and acid-base support, hyperglycemic control, and intravenous broad-spectrum antimicrobial therapy. Patients with a fulminant clinical course, unsuccessful drainage, or failed conservative therapy should undergo nephrectomy. In nondiabetic patients, successful removal of an obstruction, surgical or percutaneous drainage, and aggressive antimicrobial management may be sufficient [10, 11].

Our patient has poorly controlled insulin dependent DM and E. coli in urine cultures placing her at risk for infections including EPN. She did not have any prior instrumentation to account for a mechanical cause of emphysematous gastritis or any pulmonary issues or any ischemic source to account for gastric air. Conservative management with antibiotics, fluids and glucose control was successful in resolving the clinical and radiologic picture.

We hypothesize that our patient had emphysematous gastritis as a complication of hematogenous dissemination from emphysematous pyelonephritis.

### Conclusion

We describe a rare case of emphysematous pyelonephritis complicated with emphysematous gastritis with air in the portal vein. The air in the portal tract could be secondary to the gastric venous drainage to the portal vein. To our knowledge this is the first report showing association between emphysematous pyelonephritis and emphysematous gastritis with air in the portal vein. Our case demonstrates that immediate medical treatment with antibiotics, fluids and good glycemic control results in favorable outcomes. In addition, in diabetic patient presenting with EPN and abdominal symptoms, emphysematous gastritis should be suspected and an abdominal CT as well as an endoscopy should be considered.

## Abbreviations

CT: Computed tomogram
EPN: Emphysematous pyelonephritis
